# Personalized Nutrition for Management of Micronutrient Deficiency—Literature Review in Non-bariatric Populations and Possible Utility in Bariatric Cohort

**DOI:** 10.1007/s11695-020-04762-3

**Published:** 2020-06-20

**Authors:** Shannon Galyean, Dhanashree Sawant, Andrew C. Shin

**Affiliations:** Department of Nutritional Sciences, Texas Tech University, 1301 Akron Ave, Lubbock, TX 79409 USA

**Keywords:** Obesity, Gene, Polymorphism, Gene expression, Nutrients, Supplementation, Deficiency

## Abstract

**Background:**

Bariatric surgery can effectively treat morbid obesity; however, micronutrient deficiencies are common despite recommendations for high-dose supplements. Genetic predisposition to deficiencies underscores necessary identification of high-risk candidates. Personalized nutrition (PN) can be a tool to manage these deficiencies.

**Methods:**

Medline, PubMed, and Google Scholar were searched. Articles involving genetic testing, micronutrient metabolism, and bariatric surgery were included.

**Results:**

Studies show associations between genetic variants and micronutrient metabolism. Research demonstrates genetic testing to be a predictor for outcomes among obesity and bariatric surgery populations. There is limited research in bariatric surgery and micronutrient genetic variants.

**Conclusion:**

Genotype-based PN is becoming feasible to provide an effective treatment of micronutrient deficiencies associated with bariatric surgery. The role of genomic technology in micronutrient recommendations needs further investigation.

## Background

The prevalence of obesity is consistent with more than one third of adults having the disease of obesity [1]. Obesity is the focus for many public health efforts in the USA with one treatment option being bariatric surgery [1, 2]. Achieving weight loss is a benefit from bariatric surgery; however, micronutrient deficiencies can occur [2]. Micronutrient deficiencies are associated with serious consequences due to the negative effects on metabolic and cellular signaling pathways. Possible causes of micronutrient deficiencies after bariatric surgery are decreased food intake, food intolerance, reduced gastric secretions, bypass of intestinal surface area for absorption, as well as failure to comply with recommended vitamin regimens [3, 4]. Multiple case series have reported postoperative, malabsorptive procedures to increase prevalence of iron deficiency to 20–49%, calcium and vitamin D deficiency 25–50%, vitamin B_12_ deficiency about 33%, and folate deficiency as high as 45% [3]. For malabsorptive procedures, patients are recommended to take at least double the recommended daily dose of a multivitamin plus mineral supplement and additional 1200–2400 mg calcium, 3000 IU vitamin D to reach levels > 30 ng/mL, and vitamin B12 as needed for normal levels [5]. In a cohort of adults who underwent bariatric surgery, 73% of the patients had at least one nutritional deficiency 5 years later even though they reported taking a dietary supplement [6]. However, there are some patients, up to 47%, that may be non-responders to supplements even with compliance rates of about 86–93% [7]. Indeed, individuals respond differently to dietary interventions. Genetic variation among individuals could be the root cause for varying responses to the same regimen and explains why some individuals respond better to a certain regimen than others in the same environmental conditions [8].

## Introduction

Genetic testing can be a critical tool for health and medical diagnosis, treatment, and prevention. Predictive testing may be among the most useful tests regarding medical nutrition therapy (MNT). Genetics along with environment and behavior are the key to providing the best assessment, intervention, and tailored changes for an individual [9]. MNT should follow an appropriate paradigm that encompasses prediction (early diagnosis), prevention (intervention on healthy persons), and a tailored therapy for patients [9]. Identifying ways for early intervention may help develop strategies for preventing poor nutritional status and maximizing surgery-induced metabolic benefits later.

Sequencing of the human genome and identifying gene-nutrient interactions are the underlying concept of PN [10]. Nutrigenomics is the study of the effect of specific nutrients on gene expression [10], while nutrigenetics refers to the study of genetic variations of an individual that can provide some prediction to help prevent as well as contribute to personalized dietary management [11]. Both nutrigenomics and nutrigenetics may be a strategy to improve understanding of the gene-diet interaction and deliver individualized MNT to prevent chronic nutrition-related diseases [(10], [11)]. The usefulness and validity of this type of PN are in their infancy, although some studies have shown that individuals find dietary recommendations based on genetics more beneficial than general dietary advice [12]. A survey conducted by the publisher *Nature* showed that 27% of respondents who had their genomes analyzed changed their diet, lifestyle, or medication based on their genetic information [13]. However, another study reported that genetic testing led to no short-term changes in specific dietary or exercise behaviors [14]. Thus, increased understanding and awareness of these tests is required to effectively use them among public and healthcare providers [12].

Since many micronutrients control energy metabolism, their deficiencies can result in an array of symptoms, ranging from anemia to neurological dysfunction [15, 16]. Additionally, subclinical micronutrient deficiency can lead to increased risks for coronary artery disease, infections, age-related macular degeneration, and oxidative damage [17, 18]. Therefore, measuring nutritional status in the context of pathophysiology is critical, but this is a major challenge because it is influenced by a number of factors including dietary consumption, physical/social stressors, and infections [19]. Furthermore, the impact of nutrition could vary among individuals and specific population subgroups based on their molecular and genetic make-up [19]. Studying this complex nutrient-gene relationship to understand the metabolic networks in context of health and disease should be a focus. It can provide information on potential biomarkers of nutritional status, disease progression, and response to interventions. This literature review aims to summarize data from studies of genes involved in micronutrient metabolism. Identifying these nutrient-gene pathways and their variants can help predict those at risk for deficiencies. This may recognize the need for increasing consumption of essential nutrients to intervene prior to bariatric surgery and develop strategies to prevent micronutrient deficiency postoperatively.

## Methods

Due to the limited amount of literature published on micronutrient deficiencies, micronutrient genetic variants, and bariatric surgery, the authors conducted a narrative review. A comprehensive search of the literature from 1975 to 2020 was conducted to identify articles examining the association between genetic variants of micronutrient metabolic pathways and serum levels of micronutrients. Searches were conducted in databases that contain research related to health and metabolic outcomes, including PubMed, Medline, and Google Scholar. The search terms that were used included genetic variants, micronutrient metabolism, treatment of genetic defects of micronutrients, precision nutrition, nutrigenomics, and bariatric surgery. Additionally, review articles produced through the database searches were examined for further articles that fit within the inclusion criteria and thus were included in the results.

## Inclusion/Exclusion Criteria

Criteria for inclusion in the review were [1] peer-reviewed articles, [2] articles that included empirical data, [3] articles published or available in English, [4] articles that included people with low micronutrient levels, [5] articles that included people that had micronutrient genetic variants, [6] supplementation/treatment regimens for people with genetic variants and low micronutrient levels, and [7] articles that included genetic testing among obesity and bariatric surgery populations and outcomes that were examined. Only two articles involving micronutrient genetic variants among bariatric surgery patients were found. Due to this limitation, studies involving non-surgical patients and micronutrient genetic variants were included. Other studies involving genetic testing and bariatric surgery were included to demonstrate its potential as a tool for this patient population. There were also limitations on studies involving treatment and supplementation according to genetic variants. Ideally, inclusion criteria would comprise of studies with a high number of participants, a control group, and that used similar measures and procedures across studies for comparison; however, using the search methods and criteria described above, 80 articles met the inclusion criteria. All the authors confirmed that the articles met the inclusion criteria and were appropriate for the review. The articles that met the inclusion criteria focused on micronutrient genetic variants and genetic testing in diverse populations shifting to describe the findings.

## Results

Of the 80 articles included, 2 were published between 1975 and 1990, 5 were published between 1991 and 2000, 25 were published between 2001 and 2010, and 48 were published between 2011 and 2020. Twenty-two of these studies took place in the USA, and the rest were conducted in other countries. The articles were divided into four categories which are used to organize the results: [1] micronutrient deficiencies prevalent among bariatric surgery patients (19 articles), [2] micronutrient genetic variants prevalence among different populations (29 articles), [3] clinical trials involving supplementation for micronutrient genetic variants (26 articles), and [4] genetic testing studies in persons with obesity and bariatric surgery populations (6 articles).

### Genetic Variants and Their Effect on Vitamin and Mineral Pathways and Response to Supplementation

Genetic variations in specific genes among vitamin and mineral metabolic pathways are associated with altered nutrient homeostasis and adverse health outcomes [19]. SNPs are the most common type of genetic variations among people [20]. In the human genome, SNPs may occur at every 1000 nucleotides, which means that a person may have 4–5 million SNPs [20]. SNPs are known to impact micronutrient status or chronic diseases related to micronutrient metabolism [19, 21–23]. The ability to identify a person having genetic variants involved in vitamin and mineral metabolism may reduce the chance of developing micronutrient deficiencies that can lead to various diseases [19]. GWAS have shown that several genetic variants associated with vitamin metabolism can affect circulating vitamin levels, which could lead to abnormal vitamin function [24]. Most GWAS have been conducted among healthy, Caucasian populations, which is a limitation in this research [24]. Table 1 demonstrates recent studies that associate genetic variants and micronutrient metabolism.

**Table 1:** Relevance of genetic variants associated with micronutrient metabolism

Micronutrients	Genes identified with micronutrients	Relevance in micronutrient status	Reference
Vitamin D	1. GC 2. CYP2R1 3. DHCR7 4. CYP24A 5. VDR	1. GC gene encodes Vitamin D Binding Protein (DBP) which is a glycosylated alpha-globulin that transports vitamin D metabolites from gut and skin to target end-organs. 2. CYP2R1 gene encodes 25-hydroxylase, which converts Vitamin D to 25(OH)D. 3. DHCR7 gene provides instructions for making 7-dehydrocholesterol reductase, an enzyme involved in the final step of cholesterol production. 4. CYP24A gene provides instructions for making 24-hydroxylase, an enzyme that controls the amount of active vitamin D in the body. 5. VDR gene provides instructions for making vitamin D receptor (VDR) protein, which allows the body to respond appropriately to vitamin D >A variation in these genes may impact body vitamin D levels.	[25–28]
B12	1. FUT2 2. CUBN 3. TCN1 4. MTRR 5. TCN2 6. MTR 7. MMAA 8. MMACHC	1. FUT2 gene encodes for fucosyltransferase 2 gene and is involved in Vit B12 absorption and transport. 2. CUBN gene provides instructions for making cubilin protein which is involved in the uptake of vitamin B12. 3. TCN1 gene encodes B12-binding protein family which facilitates the transport of cobalamin into cells. 4. MTRR gene is responsible for maintaining adequate levels of activated vitamin B12, which maintains methionine synthase enzyme in its active state. 5. TCN2 provides instructions for making transcobalamin. 6. MTR gene provides instructions for making methionine synthase enzyme which needs B12 and is involved in the formation of the amino acid methionine 7. The protein encoded by MMAA gene is involved in the translocation of cobalamin into the mitochondrion. 8. It is postulated that the protein encoded by MMACHC gene may have a role in the binding and intracellular trafficking of cobalamin. >SNP related to these genes can lead to insufficient B12 levels in the body.	[28–31]
Folic acid	1.MTHFR	1. MTHFR gene produces Methylenetetrahydrofolate reductase (MTHFR) which is a vital enzyme for the folate pathway. >SNP related to this gene may be an important marker to identify people at risk for lower plasma folate concentrations, changes in folate form distribution, and elevated plasma homocysteine concentrations.	[28, 32–34]
Thiamine	1.SLC19A2 2. SLC19A3 3. SLC35F3	SLC19A2, SLC19A3 and SLC35F3 genes code for thiamine transporter protein which allow thiamine to move into the cells. >Mutations in these gene can cause thiamine deficiency leading to thiamine responsive megaloblastic anemia.	[28, 35, 36]
Iron	1.TMPRSS6 2.TFR2 3.TF 4. HFE	1. TMPRSS6 gene codes for the protein matriptase-2 which helps in regulation of iron balance. 2. TFR2 gene codes for TFR2 protein which facilitates entry of iron into the cells. 3. TF gene codes for protein transferrin which is a transport protein for iron in the body. 4. HFE gene provides instruction for production of HFE protein which determines iron absorption from diet and iron release from body stores. >A variation in these genes together has an impact on the risk of insufficient iron levels in the body.	[28, 37, 38]

### Vitamin D

Vitamin D is essential for many functions of the body. Deficiency of vitamin D is associated with many cancers, autoimmune disorders, and cardiovascular disease as well as significantly affects musculoskeletal function [39–41]. Obesity has been identified as a risk factor of vitamin D deficiency, and those seeking bariatric surgery for obesity treatment have an additional risk for low vitamin D levels post-op [39]. One study showed that 57.4% of patients seeking bariatric surgery were vitamin D-deficient preoperatively [39]. In 51 observational studies assessing vitamin D status in patients undergoing bariatric surgery, the mean (25(OH)D) level was less than 30 ng/ml (which is the minimum recommended level for optimal long-term health), before and after bariatric surgery, despite various vitamin D supplementation regimens [42]. Another review of 30 studies showed vitamin D deficiency prevalence to range from 13 to 90% preoperatively which was maintained after surgery [43].

The heritability of vitamin D status is estimated to be 30% and common variants group-specific component (*GC*) (also known as vitamin D–binding protein); 7-dehydrocholesterol reductase (*DHCR7*) and *CYP2R1* (involved in 25-hydroxylase production) are associated with fasting plasma 25(OH) D concentrations [25, 44, 45]. Nissen and colleagues have shown that 7 prominent variants in CYP2R1 and GC genes were significantly associated with low serum 25(OH) D concentrations [26]. People who have these common genetic variations could be treated on a more individualized basis to correct deficiencies that occur.

One randomized controlled trial looked at older Australians randomly assigned to monthly doses of 30,000 IU or 60,000 IU vitamin D_3_ for 12 months and found that genetic variability is associated with response to supplementation, perhaps suggesting that some people might need a higher dose to reach optimal 25(OH) D levels [46]. Another study investigated 41 candidate single nucleotide polymorphisms (SNPs) in vitamin D and calcium pathway genes among healthy non-Hispanic white participants and stated that the increase in [25(OH)D] attributable to vitamin D3 supplementation may vary according to common genetic differences in *CYP2R1*, 24-hydroxylase (*CYP24A1*), and vitamin D receptor (*VDR*) genes [27]. There is evidence from three randomized controlled trials that indicate a strong association between genetic polymorphisms and levels of serum 25(OH) D in response to 40,000 IU vitamin D/week given for 6 months [47]. However, there is a wide variation in the response of blood 25(OH) D to vitamin D supplementation that is associated with genetic variants in vitamin D metabolism [25].

### Vitamin B12

Vitamin B12 is a coenzyme, cofactor, and essential component in vitamin B complex. It is essential for cardiac health [48] and cognitive function [49, 50]. Deficiency of vitamin B12 can lead to deleterious consequences including macrocytic anemia, neuropsychiatric symptoms [51], cardiovascular diseases [52, 53], and onset of different forms of cancer [54, 55]. The most common cause of vitamin B12 deficiency is loss of intrinsic factor (IF) as absorption depends on it [56]. People who have bariatric surgery, short gut syndrome, long-term vegetarian, or vegan diets can potentially develop vitamin B12 deficiency [56]. While vitamin B12 level can be normal at baseline, it is often found to be lower in individuals after bariatric surgery [57, 58]. Nutritional parameters were compared preoperatively and at similar periods postoperatively among patients undergoing malabsorptive procedures [59]. Vitamin B12 abnormalities prior to surgery ranged from 3.2–8.3% to 24–25% at 1 year post-op [59]. In a study of gastric bypass surgery subjects, vitamin B12 deficiency was observed in 33.3% at 2 years and in 27.2% at 3 years postoperatively [60].

Genetic variants may impact the proteins involved in vitamin B12 absorption, cellular uptake, and intracellular metabolism [61–63]. Genetic influence for B12 levels is estimated to be 59% in a study using monozygotic and dizygotic twins [64] and 27% in another study among Icelandic sibling pairs [65]. Variants of the transcobalamin 1 (TCN1) gene (vitamin B12 binding protein, transcobalamin I (TCI)) have been associated with circulating B12 concentrations [29, 66]. Genetic variants of fucosyltransferase 2 (*FUT2* gene) that codes for an enzyme in the vitamin B12 pathway are associated with B12 levels [29]. Transcobalamin 2 (TCN2) gene is responsible for making a B12-binding protein called transcobolamin II (TC) that carries B12 from the intestine to blood and liver. Although TC represents approximately 10–20% of circulating B12, the most common variant of this gene among Caucasian populations has been associated with B12 levels [29]. In a study among Irish men, having this SNP and homozygous CC genotype had lower vitamin B12 levels than those with GG genotype [67]. This demonstrates that different genotypes of transcobalamin impact the distribution of vitamin B12 and shows an association between this genetic variant and B12 levels [67].

Vitamin B12 along with folate influences one-carbon metabolism. Cubulin (CUBN) is the intestinal (IF) and polymorphisms of this gene have been associated with chronic diseases in individuals with low B12 status [29]. A study involving a Canadian population found that many SNPs in genes related to folate, B12, and homocysteine metabolism—CUBN, TCN1, TCN2, methylenetetrahydrofolate reductase (MTHFR), MUT (methylmalonyl coenzyme A mutase), and FUT2—are possibly correlated with B vitamin-related diseases [30]. Genetic polymorphisms of MTHFR, MTR, MTRR, MMAA (methylmalonic aciduria (cobalamin deficiency) cb1A type), MMACHC (methylmalonic aciduria and homocystinuria, cblC type), and MUT have been analyzed. This research has failed to show an association between MTHFR gene polymorphisms and B12 concentrations [29]. However, a study using a classic twin model found that common gene variants—MMAA, MMACHC, MTRR, and MUT—were significantly associated with B12 levels and could explain the variation in B12 levels, which might facilitate the prevention and treatment of B12 insufficiency/deficiency in individuals at a higher risk of associated diseases [68]. A cross sectional study looking at 56 SNPs of the B12 pathway among an older female population and found TCN2 to be significantly associated with elevated serum methylmalonic acid (MMA) levels, a marker for available B12 [69]. When using MMA levels as a marker for B12, it is suggested that TCN2 gene variants may lead to decreased vitamin B12 availability [69]. This review spotlights the complex nature of nutrigenomics and vitamin B12. Identifying these gene variants among people having bariatric surgery could contribute to a more personalized nutrition plan.

### Folate

Folate plays a role in one-carbon metabolism, methylation and DNA synthesis, and methionine regeneration [70–72]. Folate deficiency is associated with elevated homocysteine, cardiovascular diseases, neural tube defects, cleft lip and palate, late pregnancy complications, neurodegenerative and psychiatric disorders [73–75]. Elevated homocysteine levels are a risk marker for dementia, Alzheimer’s disease, bone fractures, cancers, and cardiovascular diseases [76–78]. Many studies show folate deficiency to be low due to food fortification in America [39, 79–81]. Although preoperative deficiencies are not alarming, prevalence of folate deficiency and elevated homocysteine have shown to persist or worsen after bariatric surgery despite supplementation [82, 83]. The prevalence of abnormalities 1 year after gastric bypass were higher compared to preoperative levels in 232 patients with elevated homocysteine as high as 29% and low RBC folate in 12% of 149 postoperative subjects [82]. Another study found similar results among patients undergoing bariatric surgery with 13% having folate deficiency postoperatively [84].

Several studies have shown an association between SNPs related to folate metabolism, folate deficiency, and elevated homocysteine [70]. A common genetic variant in MTHFR is known to influence blood folate and prevalent in 10% of the population worldwide [85, 86]. Steluti and colleagues studied polymorphism frequencies and differences in homocysteine concentrations even in the presence of folic acid fortification and found that homocysteine levels increased in those carrying genetic variants in folate metabolism, specifically in the MTHFR gene [87]. The prevalence of variant MTHFR TT has been found in 25% of Americans of Hispanic origin, 10–15% among white Americans, and only 0–1% for African Americans [77, 87–89]. A review examining the nutritional deficiencies, bariatric surgery, and serum homocysteine levels found that the mutations of the MTHFR gene can be one of the reasons for persistent elevated serum homocysteine after surgery despite supplementation with B-group vitamins [76]. Knowing the presence of genetic variants of folate metabolism would provide a critical personalized care to those that might benefit from the methylated form of folic acid to prevent elevated homocysteine levels [76].

### Thiamine

Thiamine is essential for glucose, amino acid, and energy metabolisms [90–92]. Deficiency of thiamine can cause complications including cardiovascular and neurological diseases, including Wernicke-Korsakoff syndrome [90, 93]. Preoperative thiamine deficiency is prevalent in about 29% of patients undergoing bariatric surgery [57]. Studies have found that preexisting thiamine deficiency can be present in 15.5% and as high as 47% of patients; however, race plays a role showing Hispanic patients with the highest level of prevalence followed by African Americans (31%) and Caucasians (7%) [57, 79, 94]. Similarly, a retrospective study showed 33.6% of patients having thiamine deficiency pre-operatively, suggesting that people with obesity, especially those with many weight loss attempts, may have different needs to maintain adequate thiamine levels [95].

Mutations in thiamine transporter genes, SLC19A2 and SLC19A3, have been observed in cases of thiamine deficiency due to decreased absorption of thiamine that leads to neurological dysfunction [91]. SLC35F3 is another thiamine transporter gene that plays a role in cardiac health and blood pressure. Genetic variants have been associated with thiamine deficiency as well as hypertension [35]. Prevalence of mutations in these genes is largely unaccounted for despite recent advances in GWA studies. However, studies show that thiamine deficiency and cardiac dysfunction associated with these genetic variants are alleviated with thiamine supplementation [96–99]. Literature reviews have shown that treatment for thiamine deficiency vary according to the genetic defect of thiamine metabolism and that supplementation results in adequate thiamine levels and improved clinical outcomes [100, 101]. The best responses to thiamine therapy were associated with early referral for genetic testing and early initiation of thiamine treatment. This evidence demonstrates that early diagnosis of these mutations can be beneficial. It may also implicate the hereditability of thiamine deficiency and that therapeutic doses of thiamine vary according to the genetic defect.

### Iron

Iron is essential for metabolic processes like oxygen transport, deoxyribonucleic acid (DNA) synthesis, electron transport, as well as cellular functions can affect one’s well-being [102]. In individuals with obesity, the chronic inflammatory state related to obesity might be a possible risk factor for iron deficiency, which is also called the anemia of inflammation [57, 103–105]. Studies have shown that the prevalence of iron deficiency in adults with obesity is remarkable, and a decrease in serum iron and transferrin saturation levels is inversely associated with an increase in body mass index [103, 106–108]. A study involving bariatric surgery candidates showed 86.2% of females and 80% of males to be iron deficient prior to surgery [109]. A retrospective analysis of patients undergoing RYGB surgery showed that 43.9% were iron-deficient pre-operatively, which may be associated with higher complication rates as well as worsening of iron deficiency after surgery [57, 110, 111]. These findings reaffirm the need to assess and possibly intervene to manage deficiency in bariatric surgery candidates preoperatively.

Considering the results of several GWAS, there is strong evidence of genetic regulation of iron metabolism, and mutations in transmembrane serine protease 6 (TMPRSS6) gene that encodes for an enzyme that regulates hepcidin involved in iron homeostasis, iron carrier transferrin (TF), and transferrin receptor-2 (TFR2) genes have been associated with iron deficiency [112]. A GWAS concluded that identifying mutations in the TMPRSS6 gene has broad applications in understanding clinical disorders of iron metabolism, and polymorphisms in TMPRSS6 gene may contribute to iron deficiency anemia (IDA) in individuals even in absence of other predisposing factors for IDA [112]. Studies have shown a common TMPRSS6 gene variant to be prevalent in 45% of the individuals without iron deficiency and clinically relevant inflammatory conditions [104] and 36.5–41.7% in a group of non-pregnant women [113]. TF and human hemochromatosis (HFE) genes are involved in genetic regulation of maintenance of iron homeostasis [37]. Mutations in the HFE gene can lead to hereditary hemochromatosis, an iron overload disorder [114]. These factors should be considered to possibly affect iron absorption and thus response to treatment.

TMPRSS6 mutations have been associated with refractoriness to oral iron and studies confirm the role of TMPRSS6 in predicting oral iron response [114, 115]. One study evaluated subjects with persistent IDA to poorly respond to oral iron, indicating that TMPRSS6 polymorphisms are more frequent in subjects with persistent IDA [115]. Identifying mutations of these iron-related genes can help with providing personalized iron supplementation for a common deficiency post bariatric surgery.

### Association Between Genetic Defects and Micronutrient Supplementation

The vitamin and mineral supplementation studies that focus on treating genetic disorders are mainly case studies. Supplementation studies for vitamin D-related genetic variants have been conducted in populations that are overweight and have obesity. Limited data is available on micronutrient supplementation according to genetic variants in bariatric surgery populations. Table 2 shows studies involving micronutrient supplementation according to genetic defect in diverse populations.

**Table 2:** Supplementation trials according micronutrient defect

Reference	Micronutrient	Defective or mutated gene	Dosage and monitoring	No. of patients	Summary
[116]	Thiamine	SLC19A2	75 mg thiamine/day	Case study of 1 female patient	Patients with this defect present with diabetes mellitus, megaloblastic anemia, and sensorineural deafness. Thiamine supplementation improved blood glucose and insulin requirements decreased.
[117]		SLC19A3	100 mg thiamine 2×/day along with 10 mg biotin 2×/day for 5 months	Case study of 1 female patient	This genetic defect causes ophthalmoplegia, ataxia and confusion. Oral biotin and thiamine improved the symptoms dramatically the next day.
[100, 118]		TPK1	500 mg thiamine/day	2 patients with homozygous *TPK1*mutations	Early thiamine supplementation prevented encephalopathic episodes and improved developmental progression. Evidence suggests that thiamine supplementation may rescue TPK enzyme activity.
[119]	Vitamin D	GC	50,000 IU vitamin D3 per week for 8 weeks, followed by daily maintenance of 1000 IU vitamin D3 for 4 months	234 participants with vitamin D deficiency	Carriers of GC mutation showed the lowest baseline 25(OH)D levels and lowest response to vitamin D supplementation. Mutations in GC gene can predict response to vitamin D supplementation.
[27]		CYP2R1, CYP24A1, VDR	Vitamin D3 (1000 IU/day) and/or calcium carbonate (1200 mg/day elemental calcium)	1787 healthy participants	The increase in [25(OH)D] attributable to vitamin D3 supplementation may vary according to common genetic differences in CYP2R1, CYP24A1, and VDR genes.
[120]	Folic acid (FA)	MTHFR	Each treatment taken once daily for 8 weeks. 1. Enalapril only (10 mg, control group) 2. Enalapril-FA tablet (10 mg enalapril combined with 0.4 mg of FA) 3. Enalapril-FA tablet (10 mg enalapril combined with 0.8 mg of FA)	480 subjects with mild or moderate essential hypertension	MTHFR mutation can affect homocysteine concentration at baseline and post-FA treatment as well as can modify therapeutic responses to various dosages of FA supplementation.
[121]		MTHFR 677C → T genotype	3 random dietary interventions (4 months each): 1. Exclusion diet (avoidance of FA–fortified foods) 2. Folate-rich diet (folate-rich foods to achieve 400 mcg folate/d) 3. Supplement (exclusion diet plus a folate supplement of 400 mcg/day)	126 healthy subjects (42 TT, 42 CT, and 42 CC genotypes)	The TT homozygotes tended to have low plasma folate and high plasma homocysteine levels. Folate intervention on plasma folate was observed across genotypes. However, the TT homozygotes required higher supplement intervention to achieve similar effects observed in other genotypes suggesting a need for supplementation with at least 400–600 mcg/day for individuals with the TT genotype.
[122]	Vitamin B12	MTHFR 677C→T genotype	One vitamin tablet consisting of 2 mg of folic acid, 25 mg vitamin B6, and 400 μg of vitamin B12 daily for 6 months	52 patients with migraine with aura.	Vitamin supplementation lowered homocysteine and reduced migraine disability in a subgroup of patients. In this patient group the treatment effect on both homocysteine levels and migraine disability was associated with MTHFR C677T genotype; carriers of the C allele experienced a greater response compared to TT genotypes concluding that TT genotypes require a larger dosage of vitamins to exhibit the same effect as C alleles.
[123]	Iron	HFE, TMPRSS6, TF	Iron supplementation with autrin capsules (ferrous fumarate; 98.6 mg elemental iron) once a day for 20 weeks from the time of diagnosis	181 pregnant women with anemia	The HFE variant had a positive effect with significant improvement in hemoglobin, iron and ferritin. This shows an association of genetic variants and iron absorption and thus response to treatment. The TMPRSS6 mutation was significantly associated with higher serum iron and hemoglobin. The presence of variants in STEAP3, TMPRSS6, SLC11A2, SLC40A1, HAMP and TF genes indicate a probable genetic association with iron status.
[124]		TMPRSS6	Intravenous iron gluconate (1.3 mg/kg/day) for 5 days as first course and same dose was repeated after 5 months Followed by different supplementation therapy in which the patient received liposomal oral iron at a dose of 10 mg/day for 3 months	Case study of 1 female patient	A comprehensive assessment that includes sequence analysis of TMPRSS6 can help to confirm the genotype-phenotype association of genes involved in iron metabolism and may also be useful for predicting the patient’s response to iron treatment.

### Bariatric Surgery, Genetic Testing, and Gene Expression Profiles

Genetic expression patterns can be a predictive tool for responsiveness to nutritional treatments. Some studies have indicated that surgery-induced weight loss was associated with remodeling of the epigenome that helps regulate metabolic gene expression [125, 126]. One study found that 1366 genes were differentially expressed after bariatric surgery and subsequent weight loss, which are associated with gene transcription and energy metabolism [127]. Knowing the impact of bariatric surgery on the vitamin/mineral metabolic pathways can lead to successful prevention and treatment of micronutrient deficiencies. A study that specifically assessed the mRNA of genes within B12 degradation pathway after gastric bypass found that the intestine reprogrammed its genetic phenotype to compensate for the changes in B12 metabolism. The authors also found decreased expression of TCN1 but an increased production of CUBN, which reflects adaptive genetic reprogramming [128]. However, research on the role of vitamin metabolism genes and their adaptation after bariatric surgery is scarce. We do know that healthy individuals and people with obesity have different gene expression profiles and bariatric surgery further modifies the epigenome [129, 130]. Genetic testing is a useful tool for applying personalized medicine in bariatric surgery patients as demonstrated by Bandstein et al. that showed presurgery vitamin D levels may impact the size of genotype effects of FTO rs9939609 on weight loss among gastric bypass surgery patients [131]. Nutritional genomics may provide the path for precise nutrition recommendations to provide high-risk individuals with personalized treatment and to prevent micronutrient deficiencies.

## Discussion

From the review, it is evident that the deficiencies of the studied micronutrients are influenced by genetic mutations. Postbariatric surgery, patients frequently have these deficiencies and knowledge of these mutations may have bearing on its management. Additional research is needed to establish this association. This review confirmed the scarcity of research that has been conducted in the area of bariatric surgery and micronutrient genetic variants, with only two articles being found in this search. This limitation should be considered when interpreting the findings in this discussion. Furthermore, the treatment regimen for those who have micronutrient genetic variants and undergoing bariatric surgery should be an area of future research.

Personalized dietary and supplement advice derived from genetic testing should be based on appropriately designed studies. Utility of data from GWAS in providing dietary advice is limited because it is not known what diet and supplement intakes are required to prevent and treat the deficiencies that might be caused by micronutrient genetic variants. Identifying how a genetic variant modifies the response to supplementation on the micronutrient status and possibly identify responders and non-responders will be required to understand this population and area of research. Genotype along with micronutrient blood levels would be the initial step in applying PN among bariatric surgery patients. Genetic marker is only one factor that influences improvements related to micronutrient status [132].

Future work should focus on genotyping for multiple variants in the micronutrient metabolic pathways and their additive and interactive effects to get a complete understanding of the influence of genetic factors on micronutrient metabolism. Then, utilizing genomic technology to understand this influence on the responses to micronutrient supplementation is also important. This would involve micronutrient status, genetic variations, and genetic interactions within metabolic pathways involving the micronutrient, its molecular targets, and environmental stressors [133].

Furthermore, studies should focus to understand the role of the gut microbiome and its influence on metabolism and physiology. The human gut microbiota (which has its own genome) can modulate signaling pathways and regulate gene expression [134]. Diet, lifestyle, medications, and environmental exposure can increase inflammation within the gut, causing dysbiosis, which can contribute to chronic diseases and other illnesses [135]. Interestingly, gut microbial contribution to vitamin metabolism has been recognized in whole-genome metagenomic studies, suggesting microbe-mediated vitamin metabolism [136, 137]. Pre- and probiotics as well as diet can alter the gut microbiome in a manner that improves human health [138]. Investigating how the gut microbes can positively influence vitamin metabolism is warranted.

Techniques used for genetic testing will determine the cost. Methods being used in healthcare and research to identify genetic variations are known as next-generation sequencing (NGS) [28]. Sequencing costs have rapidly decreased, which has increased NGS applications in the clinical setting. There are 3 NGS approaches used: targeted gene panels (TGP), whole-exome sequencing (WES), and whole-genome sequencing (WGS) [139]. WGS implies the determination of the sequence of the entire genome of an individual; WES is a component of the genome; and TGP analyze specific mutations in a given set of genes or gene regions [140]. Per sample costs of diagnostic NGS applications, that include the total cost (in euros) of processing and analysis are estimated at €333 (TGP), €792 (WES), and €1669 (WGS) [139]. Another study showed cost analysis for cancer diagnosis using NGS. From the pre-analytical phase to delivery of results, cost per patient for TGP ranged from €376 to €968 [141]. Costs for NGS have declined and will continue to decline with innovations in genome-sequencing technologies and strategies [142]. In the area of bariatric surgery research, TGP could be utilized with the specific genes involved in micronutrient metabolic pathways, thus facilitating a more cost-effective and easy-to-interpret analysis.

Concerns and limitations involved in genetic testing include [1] the rapid growth of direct-to-consumer (DTC) genetic testing services with non-evidenced based testing, [2] potential ethical dilemmas, and [3] applications to the broader community [143]. Clinicians will be critical in providing genetic counseling regarding health decisions based on genomic information [144]. Many issues must be addressed prior to genetic testing such as informed consent, sample and data storage, return of results, and privacy and confidentiality to minimize these risks that can be introduced with such testing [145]. As mentioned previously, the majority of GWAS are conducted among Caucasian/European subjects. However, it is difficult to extrapolate from these studies to other populations. Sex differences also must be considered to improve the application of genetic tests to the broader community [143]. Addressing these areas and concerns are critical to implement genome sequencing in clinical practice. This type of analysis is likely to be cost-effective, especially in specific populations such as patients with bariatric surgery.

## Conclusion

PN is the delivery of dietary advice at an individual level and future work should verify if this targeted nutrition can change behaviors and have an impact on health outcomes. Dietitians currently provide PN advice based on diet and phenotype; however, genotype-based PN advice is not so readily available. One review, involving a variety of populations such as patients with a history of weight loss failures, people with obesity, as well as healthy men and women of various ages, examined the evidence for genotype-based personalized information on motivating behavioral change, and factors which may affect the impact of genotype-based personalized advice [146]. The researchers reported that PN advice resulted in greater dietary changes compared with general healthy eating advice [146]. Analyzing biochemical markers for vitamins/minerals as well as defining a person’s “nutrigenomic profile” for those undergoing bariatric surgery will open the door to implement more personalized recommendations for micronutrient supplementation.
